# Epigallocatechin-3-gallate induces oxidative phosphorylation by activating cytochrome c oxidase in human cultured neurons and astrocytes

**DOI:** 10.18632/oncotarget.6863

**Published:** 2016-01-09

**Authors:** Gloria Castellano-González, Nicolas Pichaud, J. William O. Ballard, Alban Bessede, Helder Marcal, Gilles J. Guillemin

**Affiliations:** ^1^ MND and Neurodegenerative Diseases Research Group, Australian School of Advanced Medicine (ASAM), Macquarie University, Sydney, Australia; ^2^ Department of Biological and Environmental Sciences, University of Gothenburg, Göteborg, Sweden; ^3^ School of Biotechnology and Biomolecular Sciences, The University of New South Wales, Sydney, Australia; ^4^ Immusmol Pty Ltd, Pessac, France; ^5^ Topical Therapeutics Research Group, School of Medical Sciences, The University of New South Wales, Sydney, Australia

**Keywords:** epigallocatechin-3-gallate, ATP, neurodegeneration, cytochrome c oxidase, mitochondria, Gerotarget

## Abstract

Mitochondrial dysfunction and resulting energy impairment have been identified as features of many neurodegenerative diseases. Whether this energy impairment is the cause of the disease or the consequence of preceding impairment(s) is still under discussion, however a recovery of cellular bioenergetics would plausibly prevent or improve the pathology. In this study, we screened different natural molecules for their ability to increase intracellular adenine triphosphate purine (ATP). Among them, epigallocatechin-3-gallate (EGCG), a polyphenol from green tea, presented the most striking results. We found that it increases ATP production in both human cultured astrocytes and neurons with different kinetic parameters and without toxicity.

Specifically, we showed that oxidative phosphorylation in human cultured astrocytes and neurons increased at the level of the routine respiration on the cells pre-treated with the natural molecule. Furthermore, EGCG-induced ATP production was only blocked by sodium azide (NaN_3_) and oligomycin, inhibitors of cytochrome c oxidase (CcO; complex IV) and ATP synthase (complex V) respectively. These findings suggest that the EGCG modulates CcO activity, as confirmed by its enzymatic activity. CcO is known to be regulated differently in neurons and astrocytes. Accordingly, EGCG treatment is acting differently on the kinetic parameters of the two cell types. To our knowledge, this is the first study showing that EGCG promotes CcO activity in human cultured neurons and astrocytes. Considering that CcO dysfunction has been reported in patients having neurodegenerative diseases such as Alzheimer's disease (AD), we therefore suggest that EGCG could restore mitochondrial function and prevent subsequent loss of synaptic function.

## INTRODUCTION

EGCG is the main polyphenol component of green tea, representing more than 10% extract in dry weight. This naturally occurring molecule is a flavonoid that belongs to the catechin subgroup. The strongest bioactivity of flavonoids is anti-oxidant which is potentiated by the catechol structure. This functional group can chemically scavenge reactive oxygen species (ROS) at relatively low concentrations [1-3] but has pro-oxidative/pro-apoptotic properties at higher concentrations [4]. Moreover, it can modulate protein functions through interactions within their hydroxyl group and the amino and carbonyl groups in proteins [5]. These properties have conferred multiple physiological and therapeutic benefits to EGCG. For example, it has been used in cancer therapy for its apoptotic and anti-proliferative properties and its activity on immune response [6-10]. EGCG has also been shown to be beneficial in autoimmune diabetes due to its anti-inflammatory activity in different cell types [11]. Finally, it has important protective effects in neurodegenerative diseases involving different molecular mechanisms and signaling pathways (Reviewed by Mandel [12]).

Although many studies attribute the neuroprotective role of EGCG to its properties as a radical scavenger, other pharmacological properties may further contribute to its therapeutic benefits. Neurodegenerative diseases are usually accompanied by fuel restrictions in neurons and mitochondrial impairment. EGCG has been previously shown to target energy metabolism in several cell types, mainly as an agonist of the main cellular energy sensor, adenosine monophosphate-activated protein kinase (AMPK) [13, 14]. Studies in mice demonstrated that EGCG can cross the blood barrier and reach the brain [15, 16] and it has been shown to accumulate in neuronal mitochondria [2]. Of interest, it has also been shown to restore mitochondrial membrane potential, mitochondrial function as well as ATP synthesis in AD mice model [1, 17].

Lower ATP production and increased formation of ROS in mitochondria are commonly observed alongside CcO impairment [18], which was shown to occur in AD pathology and other neurodegenerative diseases. CcO is the terminal oxidase of the mitochondrial electron transport system (ETS) and catalyzes the final step of the electron transfer from reduced cytochrome c to oxygen. It is also one of the proton pumps that generate the proton gradient across the inner mitochondrial membrane to power ATP synthesis. Additionally, it should be considered that CcO regulation, assembly and structure is complex and tissue specific [19].

Our results showed that EGCG induces CcO activity in human cultured neurons and astrocytes. It suggests that in addition to its antioxidant properties EGCG can induce mitochondrial respiration by activating CcO in human cultured neurons, increasing ATP production without altering redox balance. Therefore using EGCG because of its polypharmacological activities, its bioavailability properties and its restoring effect on mitochondrial function makes it a promising candidate for treating several neurodegenerative diseases with mitochondrial impairment as a common feature.

## RESULTS

### EGCG induces ATP production in human cultured neurons and astrocytes with different kinetics and dose-response patterns

After EGCG was selected for its capacity to increase ATP production in human cultured neurons (see Supplementary Figure 1), we investigated the mechanism of action of EGCG-induced ATP production by performing dose-response and kinetic studies in the two main cell types present in the brain: neurons and astrocytes.

**Figure 1:**
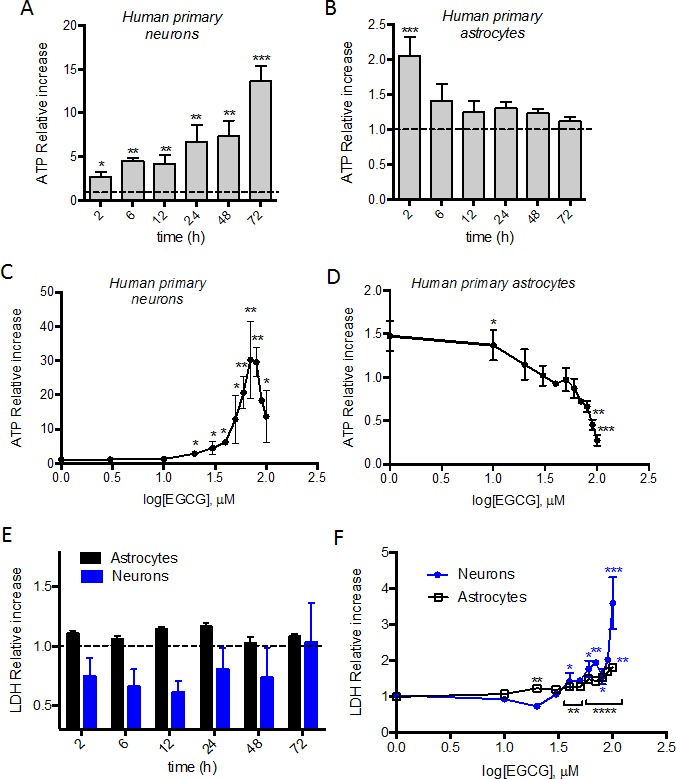
ATP modulation in neurons' and astrocytes' by EGCG treatment ATP production of neurons and astrocytes after treatment with 10μM EGCG at different time points **A**., **B**. and with increasing concentrations (1-100 μM) for 24h **C**. or 2h **D**. was assessed by luminescent intensity. Data (means ± s.e.m. of three or six experiments) are presented as normalized mean intensity in sample relative to normalized mean intensity in untreated sample for each time point. *p ≤ 0.05, **p ≤ 0.01, ***p ≤ 0.005 (treatment *versus* none; Student's *t*-test). Cell death of neurons and astrocytes after treatment with 10 μM EGCG at different time points **E**. and with increasing concentrations (1-100 μM) for 24h neurons or 2h astrocytes **F**. assessed by LDH activity in cell supernatant. Data (means ± s.e.m. of three experiments) are presented as normalized mean activity in sample relative to normalized mean activity in untreated sample for each time point. *p ≤ 0.05, **p ≤ 0.01, ***p ≤ 0.005, ****p ≤ 0.001 (treatment *versus* none; Student's *t*-test).

Both neurons and astrocytes showed a two-fold increase in ATP production after 2h treatment with 10 μM EGCG. In neurons, the ATP production is maintained and increased over 72h (Figure 1A) whereas in astrocytes the ATP is restored to basal levels after 6h (Figure 1B). Neurons and astrocytes were treated with different dose of EGCG (1 to 100 μM) for 2h in astrocytes and 24h in neurons. Increasing concentrations of EGCG (up to 60 μM) correlate with increasing production of ATP in neurons (Figure 1C). In astrocytes however, ATP production was maximal at 1-10 μM, and dropped at higher concentrations (Figure 1D). Cytotoxicity studies were done in parallel to verify that the treatments used were not causing cell death and that the increase of ATP observed was not due to induction of apoptotic pathways. Lactate dehydrogenase (LDH) was measured in the supernatant of each condition, showing no cytotoxicity over 72h in neurons and astrocytes treated with 10 μM of EGCG (Figure 1E). However, cell death was induced in neurons and astrocytes when treated for 24h with more than 30 μM and 10 μM of EGCG, respectively (Figure 1F). These results correlate with the decrease in ATP production observed in neurons (Figure 1C) at higher concentrations than 60 μM.

### EGCG increases mitochondrial membrane potential in both astrocytes and neurons

**Figure 2:**
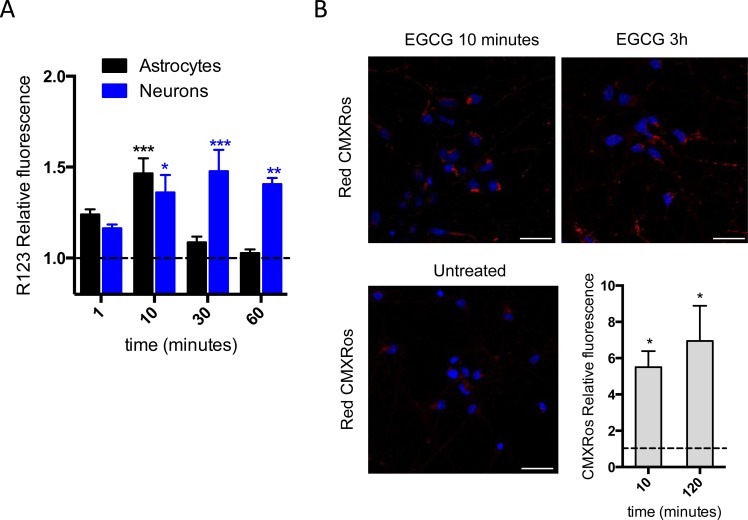
EGCG increases mitochondrial membrane potential **A**. Mitochondrial membrane potential of astrocytes and neurons assessed by R123 fluorescence intensity measured at different time points after 10 μM of EGCG treatment. Data (means ± s.e.m. from three experiments) are presented as mean fluorescence in sample relative to mean fluorescence in untreated sample for each time point. ***p ≤ 0.001, **p ≤ 0.005, *p ≤ 0.01 (treatment *versus* none; Student's *t*-test). **B**. Mitochondrial membrane potential of neurons assessed by MitoTracker CMXRos fluorescence intensity after treatment with 10 μM of EGCG. Images represent single fields from >10 fields per sample in two independent experiments. Scale bar: 25 μm. Data (means ± s.e.m. of two experiments) are presented as normalized mean fluorescence in sample relative to normalized mean fluorescence in untreated sample for each time point. *p ≤ 0.01 (treatment *versus* none; Student's *t*-test).

Mitochondrial membrane potential (Δψ_m_) relates to cells' capacity to generate ATP by oxidative phosphorylation [20]. Due to the increase in ATP production observed after 2h treatment (Figure 1A, 1B), we hypothesized that EGCG may be acting directly in one of the two main sources of ATP production in the cell: oxidative phosphorylation or glycolysis. Taking into account that the main source of ATP in neurons is mitochondrial oxidative phosphorylation [21] and that the effect of EGCG on ATP production is significantly higher in neurons than in astrocytes (Figure 1A, 1C), we therefore looked at the effect of EGCG on mitochondrial function. We examined changes on the Δψ_m_ with two different probes: R123 and CMXRos. Treatment with EGCG showed an increase in Δψ_m_ by R123 fluorescence in both cell types, reaching its maximum after 10 minutes in astrocytes and 1h in neurons (Figure 2A). Study of relative changes of Δψ_m_ was also performed using CMXRos [22] in human cultured neurons, as the increase of Δψ_m_ showed by R123 was only maintained over the time in neurons but not in astrocytes. We observed an increase on the relative fluorescence intensity of CMXRos within the neuronal mitochondria after 10 minutes treatment with EGCG (Figure 2B). Thus, the induction of R123 and CMXRos fluorescence indicates that EGCG increases Δψ_m_. Moreover, it suggests that EGCG effect on ATP production could be mediated by induction of the ETS.

### EGCG increases mitochondrial respiration in intact neurons and astrocytes but does not induce mitochondrial biogenesis

Mitochondria produce ATP *via* the electrochemical proton motive force (Δp), which is due to the transfer of electrons through the complexes of the ETS and provides the energy to drive the protons against their concentration gradient across the inner mitochondrial membrane [20]. Δp in the mitochondria is dependent of Δψ_m_ and the mitochondrial pH gradient (ΔpH) and can be represented at 37°C by the equation: Δp(mV)= Δψ_m_ −60ΔpH_m._ It should be taken into account that Δψ_m_ does not necessarily follow the proton gradient (ΔpH_m_), which is directly related to ATP production. During cellular stress, Δψ_m_ could be altered by deregulation of intracellular ionic charges (eg. Ca^2+^ or K^+^), independently of ETS induction [23]. In order to assure that the changes observed in Δψ_m_ are mediated by ETS induction, we monitored cell oxygen consumption using high-resolution respirometry.

**Figure 3:**
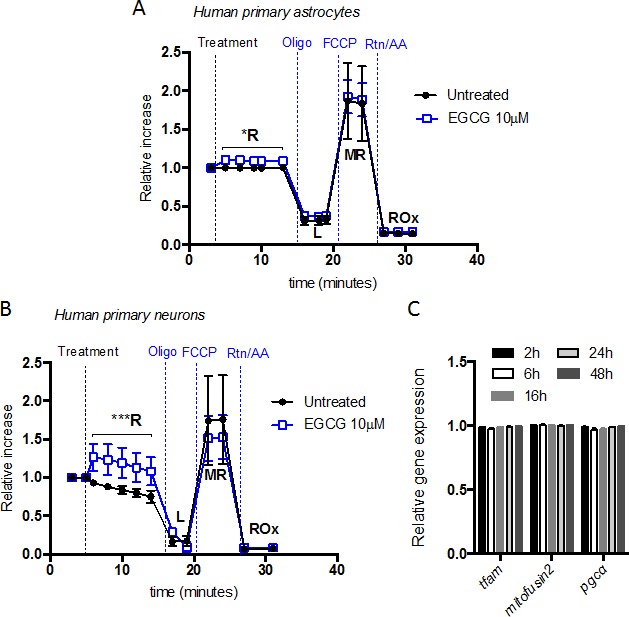
EGCG increases neuron and astrocytes routine respiration without altering mitochondrial biogenesis O_2_ consumption rate [pmol O_2_/min] of intact astrocytes **A**. and neurons **B**. showed by superimposed oxygraph traces from parallel measurements in two chambers. Treatment and mitochondrial inhibitors were added, at the time points indicated, in both chambers for the study of respiratory states. Data (means ± s.e.m. of three experiments) are presented as cell number-specific oxygen flux in sample ((pmol O_2_/mim)/10^6^ cells) relative to cell number-specific oxygen flux during routine respiration before treatment. *p ≤ 0.05 ***p ≤ 0.005 (Treatment *versus* none; Student's *t*-test). **C**. Mitochondrial biogenesis markers (*Tfam* and *PGC-α*) and mitochondrial mass marker (Mitofusin2) gene transcript expression of human primary neurons after treatment with 10 μM of EGCG at different time points. Data (means ± s.e.m. of three experiments) are presented as normalized transcript expression in the samples relative to normalized transcript expression in untreated sample (Student's *t*-test).

Considering that EGCG increases Δψ_m_ within 10 minutes, the O_2_ rate was monitored before and after EGCG addition. We found that addition of 10 μM EGCG immediately increased routine O_2_ consumption (R) in neurons (Figure 3B) and astrocytes (Figure 3A). No significant differences were observed in proton leak or ETS capacity. Cellular routine respiration is supported by exogenous substrates in the culture medium. Only physiological energy demand, energy turnover and the degree of coupling (intrinsic uncoupling and pathological dyscoupling) control the levels of respiration and phosphorylation in the physiological R of intact cells [24, 25]. Knowing that EGCG does not increase energy demand and that there are no changes on intrinsic uncoupling (L), the increase in O_2_ consumption is probably linked to an increase in energy turnover.

Measuring mitochondrial respiration in intact cells allows the integration of mitochondrial quality (function) and quantity (density). To determine whether the changes observed in routine respiration were due to an increase in mitochondrial density, we measured expression of two key genes involved in mitochondrial biogenesis, PGC-1α and Tfam as well as a marker of mitochondrial mass, mitofusin 2 [26]. Our results showed that EGCG did not influence mitochondrial biogenesis. It therefore suggests that the observed changes in mitochondrial respiration induced by EGCG were due to an increase of the mitochondrial functional properties (Figure 3C).

### EGCG-dependent ATP increase is inhibited when complex IV is blocked in neurons and astrocytes

**Figure 4:**
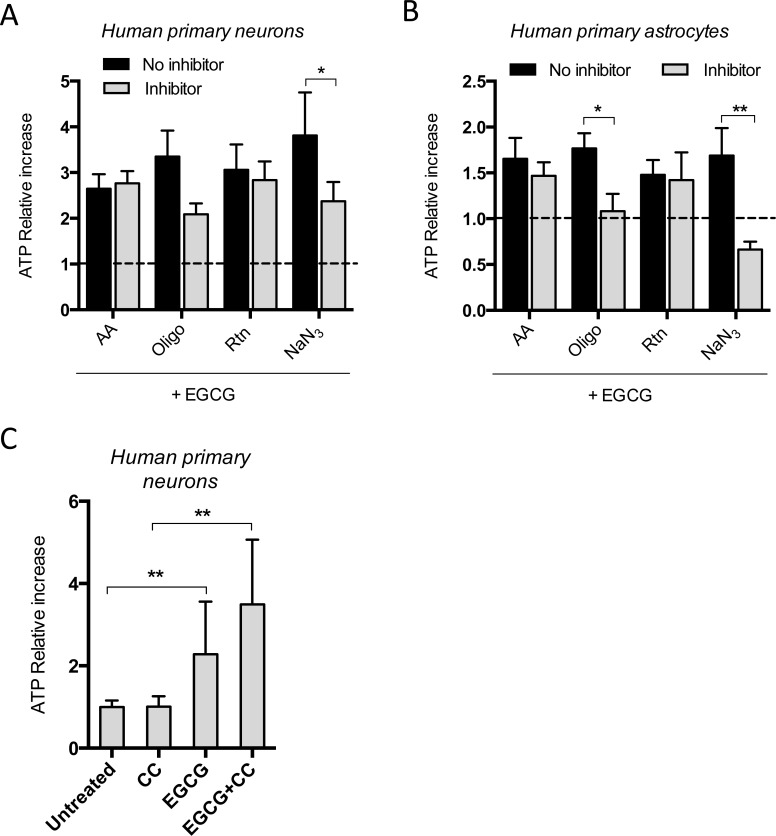
EGCG-dependent ATP increase is inhibited when complex IV is blocked in neurons and astrocytes ATP production of neurons **A**. and astrocytes **B**. after treatment with 10 μM EGCG for 16 and 2h, respectively, with or without specific mitochondrial complexes' inhibitors and assessed by luminescent intensity. Data (means ± s.e.m. of three experiments) are presented as normalized mean intensity in sample relative to normalized mean intensity in the non-EGCG treated sample from each condition (no inhibitor and inhibitor without EGCG; fold change=1). *p ≤ 0.05, **p ≤ 0.01 (Student's *t*-test). **C**. ATP quantification of neurons after treatment with 10 μM EGCG with or without CC for 16h and assessed by luminescent intensity. Data (means ± s.e.m. of three experiments) are presented as normalized mean intensity in sample relative to normalized mean intensity in untreated sample. *p ≤ 0.01, **p ≤ 0.005 (Student's *t*-test).

To establish whether EGCG enhances oxidative phosphorylation by directly activating a mitochondrial complex, we measured changes in EGCG-dependent intracellular ATP increase in the presence of different complexes' inhibitors. The time of treatments were chosen based on the previous kinetic studies, i.e. 16h in neurons and 2h in astrocytes. NaN_3_, a reversible inhibitor of complex IV, decreased the EGCG-dependent ATP production in neurons and astrocytes (Figure 4A, 4B). Moreover, when ATP synthase was irreversibly inhibited with oligomycin, a decrease in EGCG-dependent ATP production was also observed. Although neither NaN_3_ nor oligomicyn completely blocked the effect of EGCG on ATP production in neurons, their effect was stronger in astrocytes.

We also tested whether EGCG-dependent ATP production could be mediated by activation of AMPK, using the AMPK pharmacological inhibitor, compound C (CC) [27]. CC did not inhibit the EGCG-induced ATP production in neurons (Figure 4C).

### EGCG activates CcO activity in neurons without concomitant increase of ROS production

**Figure 5:**
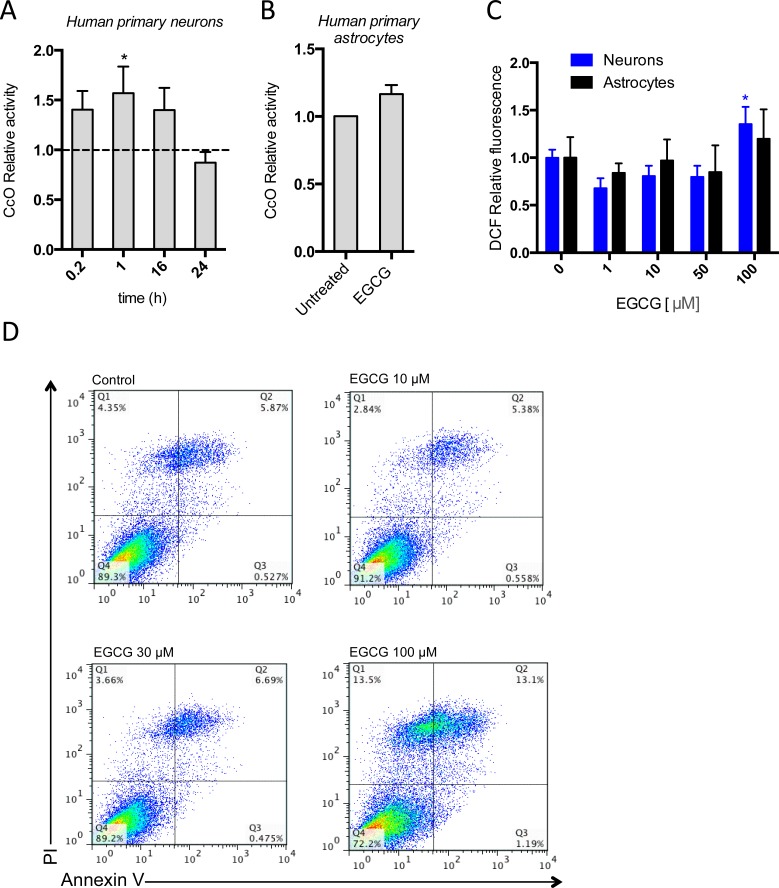
EGCG activates CcO activity without increasing oxidative stress or apoptosis CcO activity of neurons and astrocytes after treatment with 10 μM of EGCG at different time points **A**., **B**. assessed by the enzymatic degradation rate of cytochrome c and showed as relative increase of untreated. Data (means ± s.e.m. of three experiments) are presented as normalized mean activity in sample relative to normalized mean activity in untreated sample for each time point. *p ≤ 0.01 (Student's *t*-test). **C**. ROS production of neurons after treatment with different concentrations of EGCG (1-100 μM) and assessed by DCF fluorescence intensity. Data (means ± s.e.m. of three experiments) are presented as mean intensity in sample relative to mean intensity in untreated sample. *p ≤ 0.01 (treatment *versus* none; Student's *t*-test). **D**. Apoptosis of astrocytes after treatment with 10-100μM of EGCG for 48h and assessed by cytofluorometric analysis. Data are from one experiment representative of two and shown in the top right quadrant as the frequency of cells positive for Annexin-V and PI in total astrocyte population.

In order to confirm the direct effect of EGCG in CcO activity, we immunocaptured its cellular content and measured its activity by following the degradation rate of cytochrome c in cell cultures exposed to 10 μM EGCG. We observed higher CcO activity in neurons treated with EGCG compared with the controls, but the increase of CcO activity in astrocytes was not-statistically significant (Figure 5A, 5B). This confirms that EGCG is activating CcO activity in neurons, but its activation in astrocytes may not be strong enough to be detected using this method.

Molecular oxygen is reduced to water in the CcO by a sequential four-electron transfer, however a minor proportion can be reduced by a 1-electron addition that occurs predominantly in the complex III but also in the complex I [28]. High Δψ_m_ enhances the reduction of molecular oxygen to superoxide anion by complex I and III. Since EGCG is increasing CcO activity, the concomitant increase in Δψ_m_ is expected to promote higher rates of mitochondrial superoxide. In order to verify this hypothesis, we therefore measured ROS production in EGCG treated neurons and astrocytes. Treatment with EGCG up to 50 μM in both cell types did not increase ROS production, whereas 100 μM increased ROS production in neurons (Figure 5C). Excessive ROS production is associated with induction of apoptosis. We therefore measured activation of apoptosis with AnnexinV^+^/PI^−^ FACs analysis in astrocytes treated for 48h with increasing concentrations of EGCG. We did not observed increased apoptosis (AnnexinV^+^/PI^−^) or necrosis (AnnexinV^+^/PI^+^) in astrocytes treated with up to 30 μM of EGCG compared with the control. However, both necrotic and apoptotic cells were increased in 100 μM treatment with EGCG (Figure 5D). These results indicate that the induction of CcO by EGCG does not increase ROS production at the concentrations used in our study. Nonetheless, at higher doses the increase of Δψ_m_ by CcO activation could be increasing superoxide production by complex I and III, which ultimately induces apoptosis and cell death.

## DISCUSSION

The brain is highly vulnerable to energy and oxidative damage, which are the main contributing factors in the etiology of neurological disorders and ageing [29]. Therefore, restoring energy balance in the brain could be of paramount importance to improve several neurodegenerative diseases. In the current study, we demonstrated that EGCG induces energy turnover (ATP) by targeting CcO activity in the mitochondria of human cultured neurons and astrocytes. Several studies have suggested that EGCG can counteract mitochondrial dysfunction and oxidative stress mainly because of its antioxidant activity [2, 3], but to our knowledge our study is the first to show the effect of EGCG on CcO activity.

EGCG can specifically accumulate in the mitochondria of neurons and protect against oxidative stress, by acting as a natural free radical scavenger [2]. Accordingly, we observed that EGCG targets the mitochondria of two different neural cell types, inducing mitochondrial respiration. Our results are also consistent with previous studies showing that EGCG can rescue inflammatory cytokine-mediated reduction of ATP and Δψ_m_ in insulin-producing β cells [30], it can restore oligomeric Amyloid β peptides-dependent impaired ATP levels, Δψ_m_ and respiratory rates in neuroblastoma cell line and brain of AD mice model [1] and can recover mitochondria function in prion-treated neuroblastoma cell line [17]. Additionally, our data suggest that EGCG can potentiate ETS activity without inducing toxicity under physiological conditions in human cultured neurons and astrocytes.

Some studies have already linked the role of EGCG to energy metabolism as an agonist of AMPK [31-35]. AMPK is a crucial cellular energy sensor, which promotes ATP production when activated by switching on catabolic pathways while switching off anabolic or biosynthetic pathways (Reviewed by Mihayolva [36, 37]). AMPK can be activated by metabolic stresses that decrease ATP, but also through phosphorylation by Liver Kinase B (LKB) −1 complex or calmodium-dependent protein kinase kinase β (CaMKKβ). CaMKKβ is important in the neural tissue because it senses increase in cytosolic Ca^2+^, which usually trigger ATP-consuming processes [38] and could therefore be a possible target for EGCG activity. Our results show that pharmacological inhibition of AMPK in human cultured neurons with CC, which inhibits both LKB-1 and CaMKKβ-mediated AMPK activation, did not block EGCG-dependent ATP production. We also looked at the markers used to study changes in mitochondrial mass (mitofusin2) and biogenesis (PGC-1α and Tfam), which have also been found to be up regulated by AMPK [27]. AMPK phosphorylates PGC-1α, which activates its own transcription [36]. We found that EGCG did not affect mRNA levels of any of those genes, indicating that the rapid increase in ATP production is not due to activation of AMPK-mediated mitochondrial biogenesis. However, we cannot discard that EGCG acts on AMPK and activates PGC-1α [31, 39]. It is indeed possible that the fastest and first notable effect of EGCG is on ETS-ATP production resulting in an increase of ATP/ADP ratio that would be sensed by AMPK and would subsequently block its activation. In fact, when mitochondrial complex IV and V in the ETS are respectively inhibited by NaN_3_ and oligomycin, EGCG-mediated ATP production, even though reduced, is not completely blocked in human cultured neurons. This suggests that AMPK-mediated ATP production can be activated by EGCG when mitochondrial ATP turnover is blocked, thus maintaining the higher levels of ATP observed in neurons. The inhibitory effect of oligomycin and NaN_3_ on EGCG-mediated ATP production is stronger in human cultured astrocytes than in neurons. However, the variations observed between the two cell types could be due to differences in EGCG time of treatment. Longer treatment time in neurons could allow EGCG to activate secondary targets in different cellular metabolic pathways leading to ATP production. This would cover up the effects of the mitochondrial inhibitors blocking EGCG-mediated ATP synthesis through oxidative phosphorylation. Nevertheless, the divergence of metabolic regulation between neurons and astrocytes [40] should be also taken into account. Neurons main source of ATP is oxidative phosphorylation, whereas astrocytes rely on glycolysis [21]. This might explain why the EGCG-mediated ATP production *via* the ETS had a greater impact in neurons than in astrocytes under the same treatment conditions. We also observed different kinetic properties for ATP production in neurons and astrocytes, which could also be explained by differences in CcO regulation in the two cell types. CcO is composed of 13 different subunits in mammals, which are encoded by both mitochondrial and genomic DNA [41]. Many of the nuclear subunits have different isoforms, which are differently induced and expressed according to the energy requirement of the tissue (Reviewed by Arnold [19]). Among them, subunit IV is a key regulator of CcO, as it inhibits CcO when senses high ATP/ADP ratios [42]. Two isoforms of subunit IV (IV-1 and IV-2) have been described. CcO IV-1 is ubiquitously expressed in all tissues, whereas CcO IV-2 showed only high expression levels in adult lung and neurons, but not in astrocytes [43]. Neuronal CcO IV-2 abrogates allosteric inhibition of CcO by ATP, supporting a constantly high neuronal activity, whereas in astrocytes, which express CcO IV-1, ATP increase can block CcO [44, 45]. Our results show that EGCG induces an early increase of ATP in astrocytes (which resumes after 6h) as well as an exponential ATP increase in neurons (which raises over 48h). We believe that it could be explained by the ATP-mediated allosteric inhibition of CcO in astrocytes, but not in neurons, driven by the different isoforms of CcO subunit IV occurring in the two cell types. This also supports that EGCG-induced ATP increase in both cell types is mediated by an increase in ETS activity.

Another explanation for our results would be that EGCG could prevent inflammatory-induced iNOS overexpression and nitric oxide (NO) generation. It has been reported that NO can bind to the heme of CcO subunit II and inhibit its activity by competing with O_2_ [30, 46]. Therefore, EGCG could activate CcO by reducing NO-mediated inhibition of CcO. However, the induction of CcO activity by EGCG was observed under physiological conditions when there is no increase of inflammatory stress, decreased oxygen levels or alteration on CcO affinity for oxygen. It is therefore unlikely that NO inhibition participates to the EGCG mechanisms of action for CcO activation. Other important regulators, which bind to different subunits of CcO, such as the hormone sub-product 3,5-diiodothyronine [47], hypoxia-inducible factor [48] or cardiolipin [49] are known to modulate CcO activity. Alternatively, processes such as phosphorylation [50], allosteric inhibition by proton gradient [51] or availability of substrates, ADP and oxygen also participate in CcO regulation. We believe that EGCG could influence these physiological regulators and interact with them, leading to an increase of CcO activity. Another possible mechanism to take into consideration is that EGCG could act as an electron donor to cytochrome c. EGCG has a reduction energy (E_R_) of +0.43 eV and has previously been shown to be able to transfer electrons to the nucleotide deoxyguanosine monophosphate (dGMP) [52]. EGCG would act similarly to *N,N,N′,N′*-Tetramethyl-*p*-phenylenediamine (TMPD) (E_R_ =+0.23eV), which is the artificial substrate used to reduce cytochrome c in respirometry assays for measuring CcO activity. EGCG is quite stable in aqueous solution under air [52], and would not easily go under auto-oxidation, as TMPD does, therefore making unnecessary the addition of ascorbate to maintain the reduced state of the electron donor. However, further studies need to be done to elucidate the mechanistic basis of EGCG effect on the activation of CcO.

**Figure 6:**
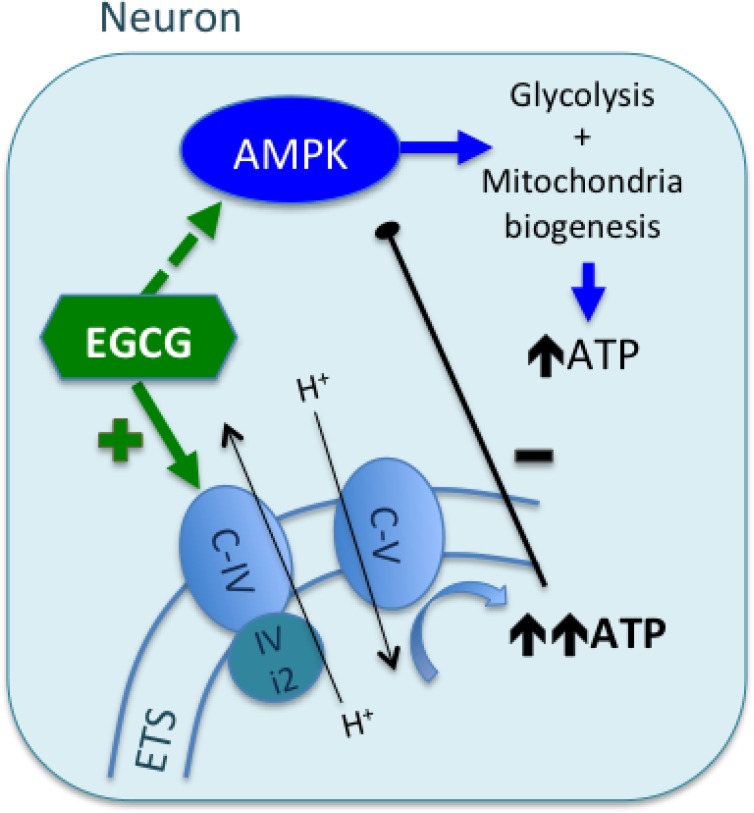
Proposed mechanism of EGCG activity in neurons EGCG can cross the BBB and reach neuronal cytoplasm where it targets mitochondrial CcO (C-IV). EGCG increases CcO activity by interacting with its physiological regulators or directly acting as an electron donor. Overall, activation of CcO leads to increase of ATP turnover by ATP synthase. High levels of ATP would be sensed by the subunit IV of CcO, allosterically inhibiting its activity. However, neurons express the isoform 2 of the subunit IV (IVi2), which is not sensible to ATP increase (differently to astrocytes, which express the isoform 1, sensible to ATP increase). EGCG can also activate AMPK, but the rapid raise of mitochondrial ATP synthesis, decreases ADP:ATP ratio, which is an inhibitory signal for AMPK activity.

EGCG has been shown to act as an antioxidant, reducing ROS induced by neurotoxic compounds [3]. In this study, we also show that used at low concentrations, it increases CcO activity and ATP synthesis without inducing ROS production, cytotoxicity or apoptosis. Specifically, our results provide compelling evidences that EGCG targets mitochondria of human cultured neurons and astrocytes, activating CcO and increasing ATP turnover without affecting the redox state of the cell (Figure 6). This natural compound may therefore be a potential interesting candidate to counteract mitochondrial dysfunction and oxidative stress observed in many neurodegenerative diseases.

## MATERIALS AND METHODS

### Cell culture

Human fetal brains were obtained from 16-19 week-old fetuses collected following therapeutic termination with informed consent. This protocol has been approved by the Human Ethics Committees of Macquarie University (Ethic approval: 5201300330). Mixed brain cell suspension was prepared using a protocol previously described by Guillemin [53]. Briefly, neurons were cultured by plating the mixed cell suspension in culture dishes or glass coverslips coated with Matrigel Matrix (1:20 in Neurobasal Medium; Corning) and maintained in Neurobasal Medium supplemented with 1% B-27 supplement, 1% GlutaMAX, 1% Antibiotic/Antimycotic, 0.5% HEPES buffer and 5mM glucose for 7-10 days at 37°C in a humidified atmosphere containing 5% CO_2_ [54, 55]. Astrocytes were obtained by seeding high density mixed brain cell suspension in a cell culture flask. Attached cells were washed with PBS and new medium was added twice per week. When the desired confluence was achieved, cells were trypsinized and expanded. Astrocytes were maintained in RPMI medium supplemented with 10% fetal calf serum (FCS), 1% GlutaMAX and 1% Antibiotic/Antimycotic at 37°C in a humidified atmosphere containing 5% CO_2_ [56-58]. All cell culture reagents were obtained from Life Technologies.

### ATP- luminescent measurements

Several natural compounds were screened for their ability to induce ATP-turnover. Treatment with kynurenic acid (KYNA) (10 μM), Phyllantus Emblica (30 μg/mL), Pomegranate (30 μg/mL), Curcuma Longa. (30 μg/mL), Berberine (30 μM) or EGCG (30 μM) (Cayman Chemical) was done for 2 and 24h in human cultured neurons. Among the different natural compounds tested, EGCG showed the most significant increase in intracellular ATP production in human cultured neurons (Supplementary Figure 1) and was therefore selected for further analysis. For EGCG-ATP kinetics induction studies, cells were treated with 10 μM of EGCG in the appropriate culture medium at different time points. For dose-dependent studies increasing concentrations of EGCG in the appropriate culture medium were added for 24h in neurons and 2h in astrocytes. For ATP depletion studies, cells were treated with 2.5 μM antimycin A (AA), 1 μM of rotenone (Rtn), 5 μM of oligomycin (Oligo), 10mM of NaN_3_ and 10 μM of CC. All reagents were obtained from Sigma-Aldrich. Treatment with the inhibitors was performed 1h before treatment with EGCG. Controls were treated with equal amount of the solvent used for each treatment. After treatments, the cells were immediately washed with warm PBS, harvested in PBS and assessed for intracellular total ATP production using a commercially available luciferase-luciferin system (ATPlite; Perkin Elmer) in a PHERAstar plate reader (BMG labtech, Ortenberg, Germany). Briefly, harvested cells were lysed by repeated freeze and thaw cycles and centrifuged at 10,000 *g* for 10 minutes; the supernatant was collected and used for ATP measurements and protein quantification. All experiments were performed using a minimum of 1mg/mL of cell protein lysate. Protein measurements were determined using Pierce BCA protein assay Kit (Thermo scientific). Total ATP was normalized against total protein to account for any difference in cell density.

### Cytotoxicity

The release of LDH in the culture supernatant correlates with the amount of cell death and membrane damage, providing an accurate measure of cellular toxicity [59]. LDH activity was measured in parallel with the ATP. It was assayed using the commercial CytoTox 96 (Promega) cytotoxicity assay following the manufacturer's specifications. LDH activity in the supernatants was normalized against total cell protein to account for any difference in cell density.

### Assessment of mitochondrial membrane potential

ΔΨ_m_ was determined using rhodamine 123 (R123; Life Technologies), as described [60], with slight modifications. Neurons or astrocytes cultures in 12 well plates were treated at different time points with 10 μM of EGCG in culture medium. Then, cells were washed with warm PBS and incubated with 10 μM of R123 (quenching mode) in Leibovitz's (L-15) Medium (Life Technologies) supplemented with 5mM of glucose for 20 minutes at 37°C in a humidified atmosphere containing 5% CO_2_. In quenching mode, the probe will accumulate within the mitochondria forming aggregates that quench some of the fluorescent emission of the dye. The R123-containing medium was removed and cells were washed with PBS and further incubated in warm L-15 Medium supplemented with 5mM of glucose at 37°C for 15 minutes. Treatment-induced mitochondrial depolarization (increase in ΔΨ_m_) results in the release of the dye, thus unquenching the dye and increasing the fluorescence signal. The sample was immediately placed in the PHERAstar plate reader at 37°C with excitation and emission set at 560 and 645nm respectively. Mean fluorescence intensity was measured using orbital averaging to account for uneven cell distribution across the well.

ΔΨ_m_ was also determined using CMXRosamine probe (CMXRos; MitoTracker Red, Life Technologies) according to previous studies showing that an increase in ΔΨ_m_ lead to an increase of CMXRos fluorescence intensity [22]. Briefly, neurons cultured in glass coverslips were treated at different time points with 10 μM of EGCG in culture medium. Then cells were washed with warm PBS prior to add 50nM of CMXRos in L-15 Medium supplemented with 5mM of glucose. Cells were incubated for 30 minutes at 37°C in a humidified atmosphere containing 5% CO_2_, then washed with warm PBS and immediately fixed with 4% paraformaldehyde (Sigma-Aldrich) for 15 minutes at room temperature. Nuclear staining was performed by incubating the cells with 4,6-diamidino-2-phenylindole (DAPI; Sigma-Aldrich) at 1 mg/mL for 1 minute at room temperature. Then, after washing with PBS, the coverslips were mounted on glass slides with Fluoromount-G (eBioscience). Epifluorescence images were obtained on an Olympus FV1000 confocal microscope (Olympus, Victoria, Australia) captured with a Charged-Coupled Device image sensor. For each coverslip, ten randomly selected fields were acquired using the same settings along each experiment. After background correction, fluorescence intensity for CMXRos was measured for each condition using ImageJ software [61]. Mean fluorescence intensity was normalized against the total number of intact cells, determined by DAPI nuclear label.

### Mitochondrial respiration in intact cells

Oxygen consumption in intact cells was measured using the high-resolution respirometer Oxygraph-2K (Oroboros instruments, Innsburk, Austria). Zero oxygen measurements were taken after injection of sodium dithionite and polarographic oxygen sensors were calibrated with air-saturated culture medium at 37°C. Intact cells were removed from the tissue culture dish with TripE (Life Technologies) and after one wash with PBS, 5 x10^6^ cells were added to 2mL of normal culture medium. Cell suspension was immediately placed in the oxygraph chamber at 37°C under continuous stirring at 300rpm. After monitoring routine respiration (R) sequential injections of the following chemicals were performed to evaluate different mitochondrial respiration rates: oligomycin (5 μM) to inhibit ATP synthase and measure the proton leak (L); FCCP (4 μM) a protonophoric uncoupler to evaluate the ETS maximum capacity (MR); and rotenone (1 μM) and antimycin A (2.5 μM) to inhibit complex I and complex III respectively and to measure the residual oxygen consumption (ROx).

### qPCR

Total RNA from cells and tissue was prepared from RNeasy Miniprep kit according to manufacturer's instructions. cDNA was prepared by using 1 μg of RNA per reaction. Standard reverse transcription (RT) was performed using SuperScript VILO cDNA Synthesis Kit according to manufacturer's instructions. qPCR analysis was carried out on the Viia7 Real-Time PCR system (Applied Biosystems, NSW, Australia) using SYBR Select Master Mix. All reagents were obtained from Life Technologies. Initial enzyme activation was performed at 95°C for 5 minutes, followed by 40 cycles of denaturation at 95°C for 5 seconds, and primer annealing/extension at 60°C for 20 seconds. Melting curve analysis was performed at 95°C for 1 minute, 60°C for 30 seconds and 95°C for 30 seconds at the end of each run to confirm a single PCR product in each reaction. The relative expression of peroxisome proliferator-activated receptor ϒ coactivator 1α (PGC-1α), mitochondrial transcription factor A (Tfam) and Mitofusin2 was normalized against the housekeeping genes TATA-binding protein (TBP) and phosphoglycerate kinase (PGK)-1. Primer sequences used for qPCR are shown in Table 1.

**Table 1:** Primers used in qPCR

Gene	Forward sequence	Reverse Sequence
Tfam	5′-CCTGCTCGGAGCTTCTCAAA-3′	5′-ACCCTTGGGGTCATTTGGTG-3′
PGC-1α	5′-AGAACAGCTAACTCCAAGTCAGATT-3′	5′-CTTCAGCTTTTCCTGCGGTGAAT-3′
PGK-1	5′-TCACTCGGGGCTAAGCAGATT-3′	5′-CAGTGCTCACATGGCTGACT-3′
TBP	5′-GGGAGCTGTGATGTGAAGT-3′	5′-GGAGGCAAGGGTACATGAGA-3′
Mitofusin2	5′-ACCCTGATGCAGACGGAAAA-3′	5′-ACCAGGAAGCTGGTACAACG-3′

### Mitochondrial complex IV content and activity

The activity of complex IV (cytochrome c oxidase) was measured by using the Mitosciences Human complex IV activity micro plate assay Kit (Abcam) according to the manufacturer's instructions. Briefly, cells were lysed and BCA assay was used to measure total protein content. The same amount of protein was loaded in each condition (≈100 μg). Complex IV was immunocaptured with CcO antibody and the activity was measured by the oxidation rate of reduced cytochrome *c* at 550 nm, using PHERAstar plate reader.

### ROS production with DCF

Intracellular oxidative stress was assessed by monitoring H_2_O_2_ (indicative of ROS generation). The amount of H_2_O_2_ can be estimated by the 2-,7-dichlorofluorescin (DCF)-H_2_ dye (Life Technologies), which oxidizes in the presence of H_2_O_2_ to its fluorescent product DCF [62]. Astrocytes and neurons were pretreated with varying concentrations of EGCG (1-100 μM) for 2h (astrocytes) and 24h (neurons). After washing twice with PBS, cells were incubated with 10 μM DCF-H_2_ in L-15 Medium supplemented with 5mM of glucose for 30 minutes at 37°C in a humidified atmosphere containing 5% CO_2_. Then, cells were washed twice with PBS and pre-warmed L-15 Medium supplemented with 5mM of glucose and 1% FCS was added. The fluorescence intensity was immediately measured at 37°C using orbital averaging in the PHERAstar plate reader with excitation and emission wavelengths set at 485 nm and 530 nm respectively.

### Apoptosis

Apoptosis was measured quantitatively using Annexin-V FITC (BD Biosciences) and propidium iodide. This protocol numerates early apoptotic cells by probing for cell surface exposed phosphatidylserine (PS) with FITC labeled Annexin-V and for plasma membrane integrity by propidium iodide (PI). Cells were treated with EGCG at different concentrations for 24h and with tumor necrosis factor (TNF)-α as an apoptotic positive control for 48h. Recovered cells were suspended in 1X binding buffer and Annexin-V FITC and propidium iodide were added prior to flow cytometry analysis.

### Data analysis

All *in vitro* determinations are presented as means ± the standard error of the mean (s.e.m.) from at least three independent experiments, unless otherwise indicated. Student's *t*-test (two-sided and paired) was used for two-group analysis, performing separate Student's *t*-test in multi-group analysis. All analyses were conducted using GraphPad Prism software (version 3.0; GraphPad Software). Statistical significance was accepted at p<0.05.

## SUPPLEMENTARY MATERIAL FIGURE


