# Supplementary material for: Epigallocatechin-3-gallate induces oxidative phosphorylation by activating cytochrome c oxidase in human cultured neurons and astrocytes
Source: Oncotarget. 2016 Jan 9;7(7):7426–40. doi: 10.18632/oncotarget.6863 (PMC4884929; doi:10.18632/oncotarget.6863)
Supplement: Supplementary file 1 [file oncotarget-07-7426-s001.pdf]

Epigallocatechin-3-gallate induces oxidative phosphorylation by activating cytochrome c oxidase in human cultured neurons and astrocytes

Supplementary Material

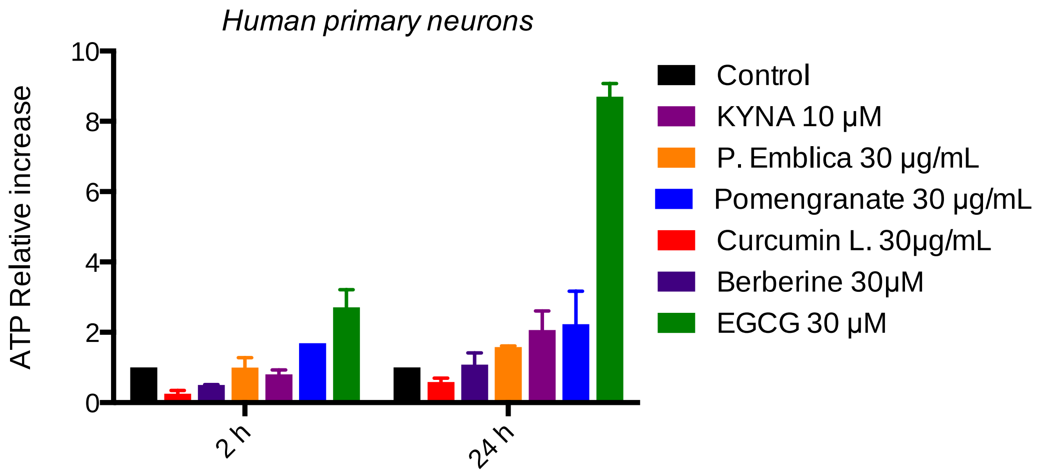

Supplementary Figure 1: Screening of natural occurring molecules for its ability to increase ATP turn over in human primary neurons

ATP synthesis in neurons after treatment with natural compounds assessed by luminescent intensity. Data (means ± s.e.m. of two experiments) are presented as normalized mean intensity in sample relative to normalized mean intensity in untreated sample. $p \leq 0.001$ (EGCG treatment versus none after 24h; Student's t-test).
